# The effect of caraway oil-loaded bio-nanoemulsions on the growth and performance of barnyard grass and maize

**DOI:** 10.1038/s41598-024-54721-6

**Published:** 2024-02-21

**Authors:** Magdalena Rys, Małgorzata Miastkowska, Anna Łętocha, Anna Wajs-Bonikowska, Paula Lorenzo, Agnieszka Synowiec

**Affiliations:** 1 The Franciszek Górski Institute of Plant Physiology, Polish Academy of Sciences, Niezapominajek 21, 30-239 Krakow, Poland; 2 Faculty of Chemical Engineering and Technology, Department of Organic Chemistry and Technology, Cracow University of Technology, 31-155 Krakow, Poland; 3 Department of Biotechnology and Food Science, Lodz University of Technology, 90-530 Lodz, Poland; 4 Department of Life Sciences, Centre for Functional Ecology (CFE)-Science for People & the Planet, Associate Laboratory TERRA, University of Coimbra, 3000-456 Coimbra, Portugal; 5 Department of Agroecology and Plant Production, The University of Agriculture in Krakow, 31-120 Krakow, Poland

**Keywords:** Biochemical changes, *Carum carvi* essential oil, Herbicidal effect, Physicochemical properties, Polydispersity index, Phytotoxicity, Environmental monitoring, Agroecology

## Abstract

A proper formulation is crucial to improve the herbicidal effects of essential oils and their selectivity. In this study, we investigated the physicochemical properties of bio-based nanoemulsions (CNs) containing several concentrations of caraway (*Carum carvi*) essential oil stabilized with Eco Tween 80, as a surfactant, maintaining 1:1 proportions. Detailed physicochemical characteristics of the CNs revealed that their properties were most desired at 2% of the oil and surfactant, i.e., the smallest droplet size, polydispersity index, and viscosity. The CNs caused biochemical changes in maize and barnyard grass (*Echinochloa crus-galli*) seedlings, however, to a different extent. Barnyard grass has overall metabolism (measured as a thermal power) decreased by 39–82% when exposed to the CNs. The CNs triggered changes in the content and composition of carbohydrates in the endosperm of both species' seedlings in a dose–response manner. The foliar application of CNs caused significant damage to tissues of young maize and barnyard grass plants. The effective dose of the CN (ED_50_, causing a 50% damage) was 5% and 17.5% oil in CN for barnyard grass and maize tissues, respectively. Spraying CNs also decreased relative water content in leaves and affected the efficiency of photosynthesis by disturbing the electron transport chain. We found that barnyard grass was significantly more susceptible to the foliar application of CNs than maize, which could be used to selectively control this species in maize crops. However, further studies are needed to verify this hypothesis under field conditions.

## Introduction

The European Union's Green Deal sets an ambitious plan to reduce the use of synthetic pesticides in European agriculture by 50% by 2030^1,2^. That results from significant issues relating to overreliance on pesticides since the 80s, mainly environmental pollution and the evolution of pesticide resistance^3,4^, but also a visible slowdown in the development of new active ingredients of pesticides in recent decades^5^. Contrary, a market based on products of natural origin that could replace synthetic pesticides, even though obstacles, is developing^6–8^. Recent research points to essential oils as possible natural compounds that can potentially replace some pesticides. For example, oils containing oxygenated monoterpenes are specifically promising as a source for botanical herbicides to control weeds at early stages of growth^9–11^. In addition, essential oils usually expose various modes of action, making it more difficult for weeds to easily develop resistance to them and being more promising to the users^12^. However, it should be noted that essential oils also have a number of structural disadvantages that limit their industrial use: volatility (influenced by heat, air, illumination, or radiation); oxidation, which may lead to the degradation of compounds that produce harm to consumers; intense aroma, reduced solubility (they are hydrophobic and incorporation in water-based matrices is challenging)^11,13^.

A proper formulation is crucial to improve the herbicidal effects of essential oils and their selectivity. Nanoemulsions are promising carrier of essential oils as they could enhance their water solubility and bioavailability, mask the unpleasant odor or taste, protect encapsulated compounds against degradation, ensure their sustainable release, and reduce the amount of active ingredient necessary for the required effect^14^. Nanoemulsions may decrease the side effects of essential oils and improve their biological activity. Due to the small droplet size and increased surface area, the nanometric formulation influences the essential oils' transport and their interaction with the multiple molecular sites at the cell membrane. Their herbicidal activity could be attributed to cell membrane disruption and increased membrane permeability^9,13,15^. Moreover, pesticides formulated with nanoemulsions use fewer organic solvents when compared with conventional formulations (emulsifiable concentrate) and have lower surfactant concentrations (between 3 and 10%) than microemulsions (even up to 40%). Compared to the conventional emulsions, the small droplet size of nanoemulsions presents additional advantages such as greater spreadability, wettability, and superior mechanical stability. The low viscosity of nanoemulsions is also important for the formulation of bioherbicides, allowing them to be applied on plants as an aerosol. Consequently, nanoemulsions incorporating pesticides settle uniformly on the plant leaves^14^.

Our previous research found that peppermint oil-loaded nanoemulsion stabilized by bio-based ethoxylated sorbitan ester of oleic acid (Eco Tween 80) and sorbitan oleate at 2% concentration can be used as a perspective preparation for selective control of barnyard grass in maize^16^. The present study used the same preparation procedure, but with caraway essential oil as the active ingredient. The caraway oil is distilled mainly from the fruits of annual or biannual spice caraway (*Carum carvi* L.) from the Apiaceae botanical family. Caraway is an herbaceous plant native to Europe, Asia, and Africa. Caraway fruits (*Carvi Fructus*) are spices in bread, casseroles, curry foods, and other foods and are also a flavoring agent in some cheeses, aquavits, and liqueurs^17^. Ten tones of caraway essential oil are produced annually, mainly in Egypt and the Netherlands. The average oil content from the European populations of caraway varies between 3.2 and 5.2%^18^. The main components of the caraway oil are cyclic monoterpenes: oxygenated terpene l-carvone (isomer: S-(+)) (50–70%) and nonoxygenated terpene d-limonene (isomer: R-(+) (40%)^19,20^, which comprise over 95% of the total oil composition^21,22^. The caraway oil displays perspective pesticidal properties and was studied as a botanical insecticide^23–25^ and also as a botanical herbicide against germination of selected weeds and crops in laboratory conditions^10,26–28^. The caraway oil was also used as an active ingredient in oil-in-water emulsions with fatty acid methyl esters^29,30^, which were applied on maize and barnyard grass leaves, causing severe leaf necrosis and reduction in biomass as well as significant alterations in the photosynthetic apparatus and plant metabolism.

Our previous research^16,31–36^ has highlighted three non-destructive methods that are essential in studying plant responses to phytotoxic stress caused by herbicides or essential oils. These methods include: measurements of chlorophyll *a* fluorescence showing the efficiency of the photosynthetic apparatus, Fourier-transformed (FT) Raman spectroscopy allowing the examination of the chemical composition, and heat flow measured by microcalorimetry assessing the viability of seedlings.

Chlorophyll *a* fluorescence is a widely used method to assess the physiological status of plants and allows detecting the impact of stress conditions such as drought, high and low temperature, nutrient deficiencies or pathogen infections at early stress stages, when other symptoms are not yet visible.. This technique measures the light emitted by chlorophyll molecules when they return to their ground state after being excited by light, which can be expressed by changes in several fluorescence indices^37–40^.

FT-Raman spectroscopy relies on the inelastic scattering of light by molecules. When a sample is irradiated with monochromatic laser light, some photons are scattered at different wavelengths due to molecular vibrations. The resulting Raman spectrum provides information about the vibrational modes of molecular bonds, allowing the identification and quantification of different chemical compounds in the sample. Plants respond to stress by undergoing biochemical changes at the molecular level. These changes can be detected and characterized by studying the Raman spectra of plant tissues. Different cellular components, such as lipids, proteins, carbohydrates, and nucleic acids, exhibit characteristic Raman bands that can be monitored to assess stress-induced alterations^35,41^.

Microcalorimetry involves studying the heat changes associated with metabolic activities and cellular responses in plant tissues under stress conditions. Microcalorimetry can gain a deeper understanding of the energy-related aspects of plant metabolism and how these processes are affected by external stressors^31,33,35^. In this study, we investigated i) the physicochemical properties of bio-based nanoemulsions with caraway essential oil and ii) their herbicidal and biochemical effects on seedlings and young plants of maize and barnyard grass.

## Materials and methods

All methods presented in this manuscript were carried out in accordance with relevant guidelines, which are cited in the particular sectionsdescribing each method.

### Chemical identification of caraway essential oil and description of tested model plants

The caraway essential oil was purchased from Avicenna Oil company (Wroclaw, PL). The content of the main compounds of the oil, carvone, and limonene was analyzed by a gas chromatography coupled with mass spectrometry and flame ionization detector GC–MS–FID, according to the method described by Rys et al.^16^. The analyses were performed on a Trace GC Ultra coupled with a DSQII mass spectrometer (Thermo Electron, Waltham, MA, USA). A simultaneous GC-FID and MS analysis was performed using an MS-FID splitter (SGE Analytical Science, Ringwood Victoria, Australia). The mass range was 33–550 amu, with an ion source heating of 200 °C and an ionization energy of 70 eV. One microliter of caraway oil (80% v/v) diluted in pentane: diethyl ether was injected in split mode at split ratios (50:1). Operating conditions for capillary column Rtx-1 MS (60 m × 0.25 mm i.d., film thickness 0.25 μm), and temperature program: 50 °C (3 min)–300 °C (30 min) at 4 °C ^.^min^−1^. Injector and detector temperatures were 280 ˚C and 300 ˚C, respectively. The carrier gas was helium (constant pressure: 300 kPa). Operating condition on a Chirasil-Dex CB column (Agilent, Santa Clara, CA, USA) having the following dimensions: 30 m × 0.25 mm i.d., 0.25 xm df. Temperature program: 50 (3 min)–220 °C (30 min) at 4 °C min^−1^. Injector and detector temperatures were 240 °C and 250 °C, respectively. The carrier gas was nitrogen at a 1.0 mL min^−1^ flow rate^16^.

The percentages of carvone and limonene were computed from the GC peak area without using a correction factor. The identification was based on comparing their mass spectra with those in Adams^42^ and computer libraries: NIST 2012, and Wiley Registry of Mass Spectral Data 8th edition, along with the relative retention indices (RI, non-polar column). Identifying enantiomers within the main caraway oil volatiles was based on the enantiomeric standards of these terpenes (l- and d-).

The effect of nanoemulsions with caraway oil was tested on two plant species. A model crop was maize [*Zea mays* L., cv. Lokata)] obtained from a breeder HR Smolice, PL. A model weed was barnyard grass [*Echinochloa crus galli* (L.) P. Beauv.], whose seeds were collected in the summer of 2019 at a full panicle maturity from a neighboring field population in Mydlniki/Krakow (50°04′48" N 19°50′58" E). The plant material was collected respecting the IUCN policy^43^. Based on Rutkowski^44^, the specimens were botanically identified by Dr. A. Synowiec and then archived in the herbarium resources of the Department of Agroecology and Crop Production of the University of Agriculture in Krakow, which provides access to deposited material (voucher number KAiPR-2019-01).

### Preparation and physicochemical characteristics of caraway oil nanoemulsions

Nanoemulsions consisted of caraway oil, Eco Tween 80 (Croda Poland) as surfactant and deionized water. The emulsifier was selected because it is 100% bio-based ethoxylated sorbitan ester based on a natural oleic acid. All formulations and physicochemical analyses were prepared and conducted according to the methodology described in our previous paper^16^. The first step was the preparation of the pre-emulsion mixture by combining the aqueous phase with the caraway oil and surfactant at Temperature (T) ≤ 40 °C, under magnetic stirring (v = 300 rpm). Then, as a second step, the coarse emulsion was processed with a probe-type sonicator (UP200 Ht, Hielscher) with a maximum power output of 200 W.

The base nanoemulsion recipe was defined using the statistical method tool for designing experiments with a fractional plan of ${3}^{(K-p)}$, where K is the number of variables and p always takes the value 1. It was generated in the Statistica^®^ ver. 13 software (TIBCO Software Inc., Palo Alto, CA, USA).

Table 1 presents the specific values of each process parameters as well as the analytical results.

**Table 1:** Matrix of the experimental design and experimental data obtained for the dependent variables.

Sample	Input parameters				Output parameters			
	C_Oil_ (%)	C_Emulsifier_ (%)	Amplitude (%)	Time (min)	Z-Ave (d.nm)	PDI	Viscosity (mPa∙s)	Stability
N17	3	5	79	3	128.2	0.11	207.543	+
N13	3	3	69	2	20.86	0.393	178.252	+
N18	3	5	89	2	18.87	0.364	206.077	+
N23	5	3	79	3	1215	0.547	–	−
N6	1	3	89	1	84.58	0.191	170.124	+
N16	3	5	69	1	132.8	0.099	249.108	+
N20	5	1	79	1	226.1	0.273	116.367	+
N21	5	1	89	3	341.1	0.51	135.574	+
N4	1	3	69	3	219.8	0.839	–	−
N14	3	3	79	1	216	0.999	–	−
N26	5	5	79	2	125.5	0.109	153.062	+
N22	5	3	69	1	413.6	0.277	361.335	+
N8	1	5	79	1	383.1	0.398	125.844	+
N9	1	5	89	3	164	1	–	−
N25	5	5	69	3	153.9	0.154	373.038	+
N10	3	1	69	3	113.5	0.072	145.612	+
N11	3	1	79	2	156.1	0.167	116.367	+
N5	1	3	79	2	16.8	0.333	168.54	+
N12	3	1	89	1	91.78	0.057	200.646	+
N7	1	5	69	2	168.6	0.122	321.997	+
N2	1	1	79	3	313.2	0.482	148.69	+
N15	3	3	89	3	6015	0.501	–	−
N24	5	3	89	2	171.7	0.092	137.194	+
N3	1	1	89	2	298.1	0.368	146.762	** + **
N27	5	5	89	1	235.8	0.219	227.419	** + **
N1	1	1	69	1	224.8	0.233	174.613	+
N19	5	1	69	2	171.8	0.293	232.1	+
N28	3	3	79	2	1215	0.547	207.543	** + **

*C* concentration, *Z-Ave* average droplet size of nanoemulsions, *PDI* polydispersity index, ( +) the sample passed stability tests and its size did not change with time, (−) the sample did not passed stability tests and destabilized.

The nanoemulsions' mean droplet diameter and polydispersity index were measured with a Dynamic Light Scattering (DLS) method (Zetasizer Nano ZS, Malvern Instruments, Malvern, UK) at 25 °C. The rheological properties of the obtained preparations were determined with an R/S rotational rheometer with cone/plate measuring elements (cone C25-1) at room temperature (25 °C). Viscosity tests were conducted with a variable cutting rate within the 1–500 r.p.s. Stability tests of the obtained samples were examined with the centrifuge method, the test of variable temperatures, and the examination of the size change over time.

To conduct bioassays we selected nanoemulsions with positivity stability tests (Table 1).

### Metabolic activity of maize and barnyard grass seedlings treated with different concentrations of caraway oil nanoemulsions

Metabolic activity during the growth of maize and barnyard grass seedlings in the presence of caraway oil nanoemulsions (CNs) was measured at 20 °C using an isothermal calorimeter TAM III equipped with TAM Assistant Software (Thermo Activity Monitor, TA Instruments, New Castle, DE, USA) according to Rys et al.^16^. Briefly, ten maize kernels and 50 barnyard grass seeds were placed on filter paper moistened with 5 mL (for maize) and 2 mL (for barnyard grass) of distilled water and allow to germinate at 21 °C in darkness. Three two-day-old maize seedlings were transferred into a 25 mL calorimetric ampoules with 400 μL of CN1-4 (caraway oil concentration: 1%; 1.5%; 2% or 5%), or surfactant (S1—1% or S5—10%), or distilled water, at the bottom at 20 °C in darkness. The reference ampoule contained 400 μL of CN in an appropriate concentration or water (control) only. For barnyard grass, five 7-days-old seedlings were placed into 4 mL calorimetric measuring ampoules with 40 μL of CN1–4 (oil concentration: 1%; 1.5%; 2% or 5%), or surfactants (S1—1% or S5—10%) or water at 20 °C in darkness. Ampoules containing only 40 μL of 1% surfactant, 10% surfactant or distilled water were controls. Treatments were replicated six and fifteen times for maize and barnyard grass, respectively. Thermal power curves were recorded for 24 h in each replicate for each treatment. The thermal energy value was calculated by integrating the relevant thermal power curves as they equal the area under these curves.

### FT-Raman spectroscopy of maize and barnyard grass seedlings treated with different concentrations of caraway oil nanoemulsions

Seedlings from the previous assays were lyophilized and stored at −80 °C until use. The Raman spectra of lyophilized seedlings were recorded in the endosperm of kernels cut in half using a Nicolet NXR 9650 FT-Raman Spectrometer (Thermo Scientific, Walthman, MA, USA) equipped with an Nd: YAG laser (1064 nm) and an InGaAs detector. The measurements were performed according to the method described by Troć et al.^45^ at room temperature at a spectral resolution of 8 cm^−1^ using an unfocused laser beam approximately 50 μm in diameter and an aperture of 80 μm. The laser power was 0.5 W, and the measurement range was 400 to 2000 cm^−1^. For each object 64 scans per spectrum were performed. The Raman spectra were registered and processed using the Omnic/Thermo Scientific software program (Thermo Scientific, Walthman, MA, USA). Six spectra from different plants were collected and averaged for each plant species and treatment. The spectra were baseline corrected. A hierarchical cluster analysis (similarities between FT-Raman spectra) was used to group the studied objects into clusters to find significant and systematic differences in the measured FT-Raman spectra and was performed using Statistica ver. 13.3 (TIBCO Software Inc., Palo Alto, CA, USA) for the whole wavenumber range. The spectral distances were calculated using Ward's algorithm.

### Herbicidal effects of leaf-applied caraway oil nanoemulsions of different concentrations on maize and barnyard grass

In a randomized design, a pot experiment with four replications per treatment was carried out in a naturally ventilated vegetation hall with natural access to sunlight in spring 2021 at the experimental station of University of Krakow, Poland. 0.5-L plastic pots (11 cm diameter) were filled with local sandy soil of pH 5.7 that was sieved (sieve size 2 cm) to remove any impurities. Two kernels of maize or ten seeds of barnyard grass were sown in separate pots on June 2, 2021. After the emergence of barnyard grass, the seedlings were thinned to five per pot. Pots were watered according to their needs. On June 21st, 2021, plants at the three-leaf stage were hand-sprayed with a dose of 0.02 L m^−2^ of either CNs (1%, CN1; 1.5%, CN2; 2%, CN3; 5%, CN4; 10%, CN5), or surfactant (1%, S1; 10%, S5), or distilled water (C, control) or a commercial mixture of herbicide (H) foramsulfuron + iodosulfuron-methyl sodium + thiencarbazone methyl (39.4 + 1.25 + 12.5 g ha^−1^; MaisTER Power 42.5 OD, Bayer CropSci, PL).Seven days after spraying, necrosis of aboveground tissues was visually estimated on a percentage scale (0–100%).

#### Chlorophyll *a* fluorescence of maize and barnyard grass leaves following nanoemulsions spraying

To assess the efficiency of photosystem II, the chlorophyll *a* fluorescence measurements were performed using a Plant Efficiency Analyser (PEA, Hansatech Ltd., Pentney, UK). The measurements were taken 72 h after spraying maize and barnyard grass, in the central part of mature leaves, which were adapted to the dark for 30 min using special clips^31^. The following parameters of phenomenological energy fluxes were calculated from the fluorescence curve: the energy absorption by the antenna pigments (ABS/CS_m_), the amount of energy that was trapped in the reaction center (TR_0_/CS_m_), the energy flux for the electron transport (ET_0_/CS_m_), and the dissipation of energy as heat (DI_0_/CS_m_) where CS_m_ is the sample cross-section. The same parameters were calculated for the reaction center (RC) and named specific energy fluxes. Moreover, the maximum quantum yield of the photosystem II primary photochemistry (F_v_/F_m_ ratio), the changes in heat dissipation in the PSII antenna (F_v_/F_0_), maximum quantum yield for primary photochemistry [Phi (Po)], quantum yield for electron transport (ET) [Psi (Eo)], as well as the PSII performance index (PI) were calculated. The detailed equations for the specific parameters are based on Strasser et al.^37^. The measurements were performed in 15 biological replicates for each plant species and treatment (one biological replicate—one individual leaf).

#### Relative water content in leaves of maize and barnyard grass following nanoemulsions spraying

Relative water content was assessed according to Rys et al.^16^. 72 h after spraying plants, leaf fragments of maize and barnyard grass (approximately 20–30 mg) were cut from the central part of a fully developed leaf and weighed (fresh mass—FM). Next, the leaf fragments were placed in separate vials with 50 mL of water and shaken (WL-972, JW Electronic, Warsaw, Poland) at 20 °C for 24 h. Then, the leaf fragments were weighed again to determine the turgid mass (TM) and dried for 24 h at 105 °C to determine the dry mass (DM). The relative water content (RWC) was calculated using the equation:

1 $${\text{RWC (}}\% {) } = \, \left( {({\text{FM }} - {\text{ DM}})/({\text{TM }} - {\text{ DM}})} \right) \, \times { 1}00\% ,$$

where: FM is the fresh mass; DM is the dry mass; and TM is the turgid mass.

The results are the mean value of ten replicates for barnyard grass and eight replicates for maize for each treatment.

### Statistical analyses

Statistical analysis of the caraway oil-loaded nanoemulsions (CNs) physico-chemical properties was performed based on a one-way analysis of variance (ANOVA). The normality of dependent variables was checked before conducting ANOVA. The significance of the differences was evaluated using the F-test. It was checked whether the input parameters significantly affect the output parameters of the CNs. As in case of our previous studies^16^ the input parameters included oil concentration [coil (%)], the emulsifier concentration [cemulsifier (%)], the amplitude, and the sonification time. The group of initial parameters included the particle size (nm), polydispersity index (PDI), viscosity (mPa s), and stability. A value of *p* < 0.05 was established as significant. Given the profiles of the utility function to certain independent parameters, thanks to which it was possible to determine changes, *p* < 0.05 was established as significant in all the CNs.

Physiological data from the calorimetric measurements, pot experiment, chlorophyll fluorescence and RWC were analyzed by one-way ANOVA, and means were separated by Duncan's posthoc test at a significance level of *p* ≤ 0.05 in Statistica ver. 13.3 software (Tibco Software Inc., Palo Alto, CA, USA).

The percentage scale (0–100%) of tissue necrosis after the CNs1-5 leaf-spraying was used to estimate the effective doses (ED), causing 10%, 50%, and 90% damage to maize and barnyard grass. The ED values were calculated using the concentration–response log-logistic analysis with a four-parameter curve according to the following equation:

2 $${\text{y}} = {\text{d }} + \, \left( {{\text{a}} - {\text{d}}} \right)/({1} + \left( {{\text{x}}/{\text{c}}} \right)^{{ - {\text{b}}}} ),$$

where: x is the dependent variable; y is the independent variable; a is the minimum value that can be obtained; d is the maximum value that can be obtained; c is the point of inflection; b is the Hill's coefficient (e.g. the negative slope at the inflection point). The calculations were carried out in the *drc* package^46^ in the RStudio 2022.02.0 Build 443 software (Prairie Trillium, USA).

## Results

### Characteristic of main compounds of caraway essential oil

Thirty-one volatiles were identified in caraway oil, constituting 99.7% of all detected compounds. The main compounds found in the oil were carvone (l-isomer) (63.3%) and limonene (d-isomer) (35.2%), accounting for a total of 98.5% of caraway oil. β-myrcene, cis- and trans-dihydro carvone constituted 0.2 and 0.3% of the oil, respectively.

### The influence of input parameters on physico-chemical properties of nanoemulsions

Based on the results from the design of experiments tool, samples of nanoemulsions containing caraway oil were prepared. All samples were prepared using the minimum ultrasonication time (t = 1 min) and the maximum amplitude value (89%). Stability analysis showed no significant changes in droplet size and polydispersity (Table 1). Moreover, all the samples are characterized by a very small droplet size of around 100 nm and very low viscosity values in the range of 170 – 360 mPa∙s, which are desired values of the output parameters (Table 2).

**Table 2:** Physicochemical properties of selected nanoemulsions containing different caraway oil concentrations.

Nanoemulsion (% of emulsifier)	Z-Ave (d.nm)/PDI after 24 h	Z-Ave (d.nm)/PDI after stability tests	Viscosity (mPa∙s)
CN-1 (1.0)	117.4 ± 2.3/0.158 ± 0.009	107.0 ± 0.7/0.146 ± 0.007	174 ± 10
CN-2 (1.5)	76.8 ± 0.8/0.131 ± 0.011	84.0 ± 1.5/0.096 ± 0.007	207 ± 12
CN-3 (2.0)	68.1 ± 0.7/0.137 ± 0.007	72.9 ± 1.4/0.116 ± 0.019	315 ± 4
CN-4 (5.0)	80.1 ± 2.2/0.136 ± 0.008	84.5 ± 1.3/0.070 ± 0.002	360 ± 7

*Z-Ave* average droplet size, *PDI* polydispersity index.

**Figure 1:**
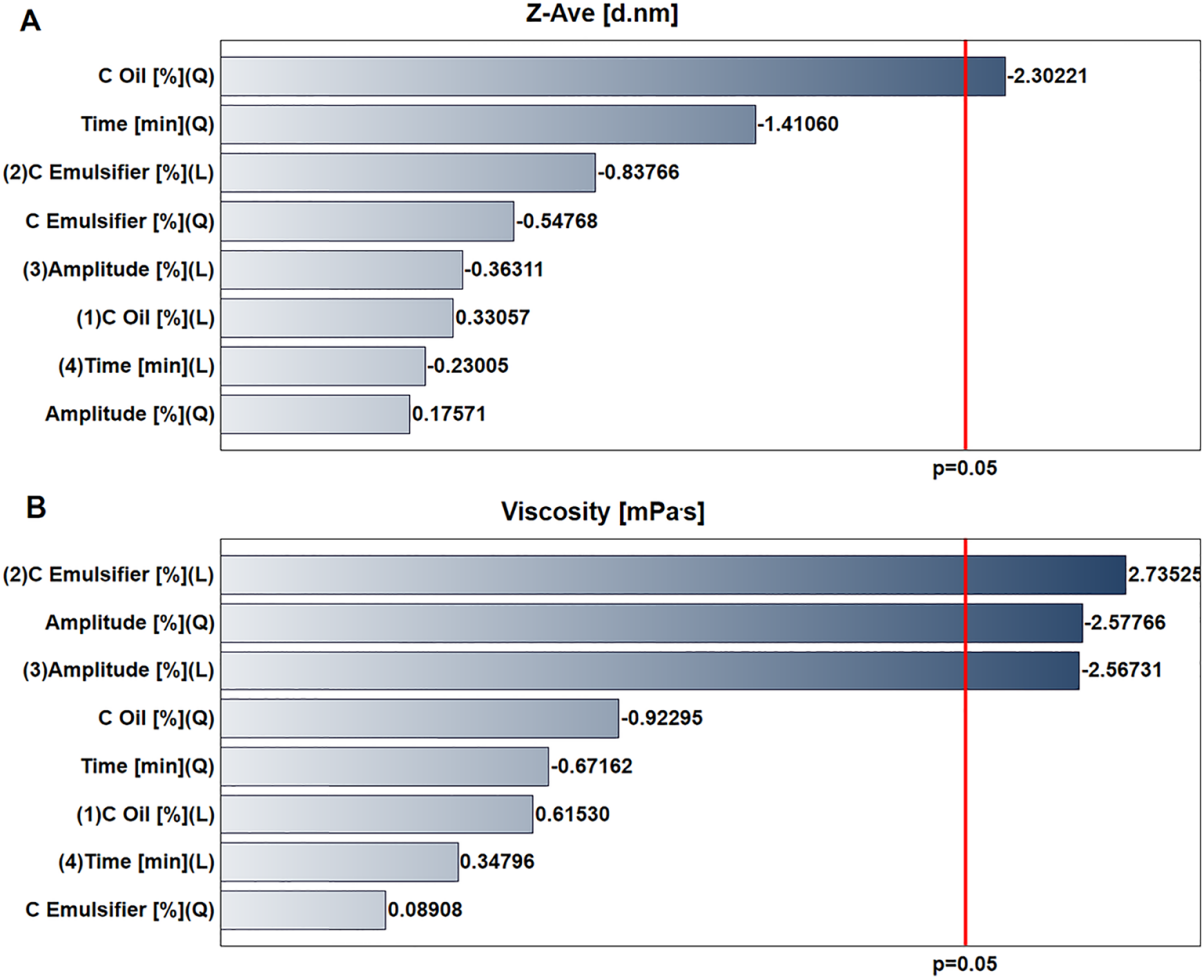
Pareto charts for the influence of input parameters on (**a**) average droplet size of nanoemulsions (Z-Ave, d.nm), (**b**) viscosity (mPa s). Parameters statistically significant are marked with a red line (*p* < 0.05).

The oil concentration had a statistically significant impact on the particle size of nanoemulsions (Fig. 1a). By contrast, the concentration of the emulsifier (linear function) and amplitude (linear and quadratic function) significantly influenced viscosity (Fig. 1b). Neither the polydispersity index (PDI) nor the stability depended on any of the input parameters analyzed (data not shown).

**Figure 2:**
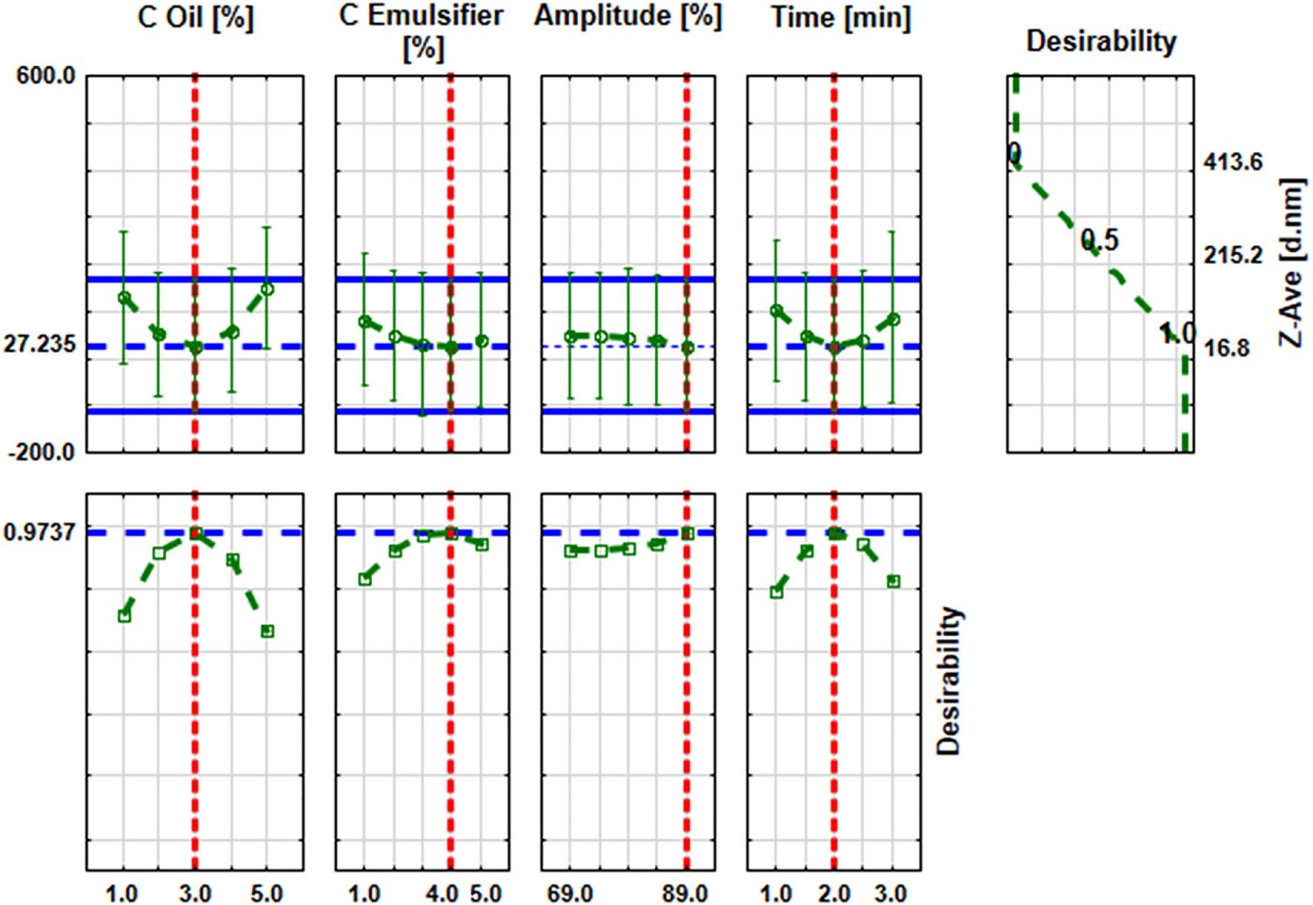
Approximation profiles for the influence of input parameters on average droplet size of nanoemulsions (Z-Ave, d.nm).

The analysis of the approximation profiles (Fig. 2) showed that the nanoemulsions with the smallest particle size (approximately 27 nm) are obtained for an intermediate oil concentration of 3%, the emulsifier concentration of 4%, the ultrasonication time = 2 min and the amplitude value (89%). Figure 2 also showed that in the case of oil and emulsifier concentration and sonification time, the particle size decreased to the intermediate values of those input parameters and then began to increase. In the case of amplitude, the sizes decreased with increasing ultrasound amplitude.

**Figure 3:**
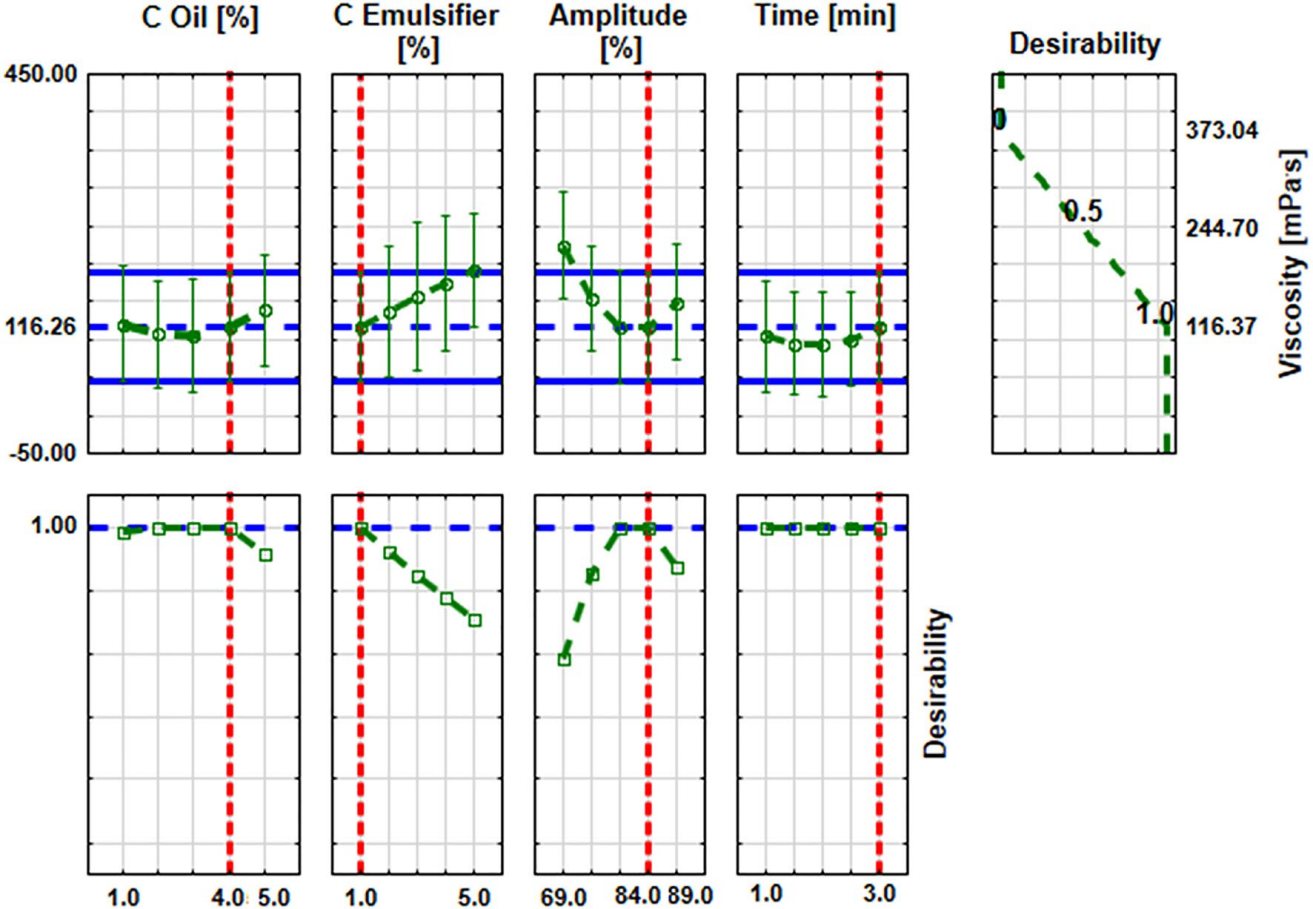
Approximation profiles for the influence of input parameters on viscosity of nanoemulsions (mPa s).

The analysis of the approximation profiles showed that nanoemulsions with the smallest viscosity (116 mPa s) were obtained with an oil concentration of 4%, an emulsifier concentration of 1%, an ultrasonication of 3 min and an amplitude value of 84% (Fig. 3). This figure also showed that the viscosity increased with increasing emulsifier concentration. In the case of oil concentration and amplitude, the viscosity decreased to 4% and 84%, respectively, and then started to increase.

**Figure 4:**
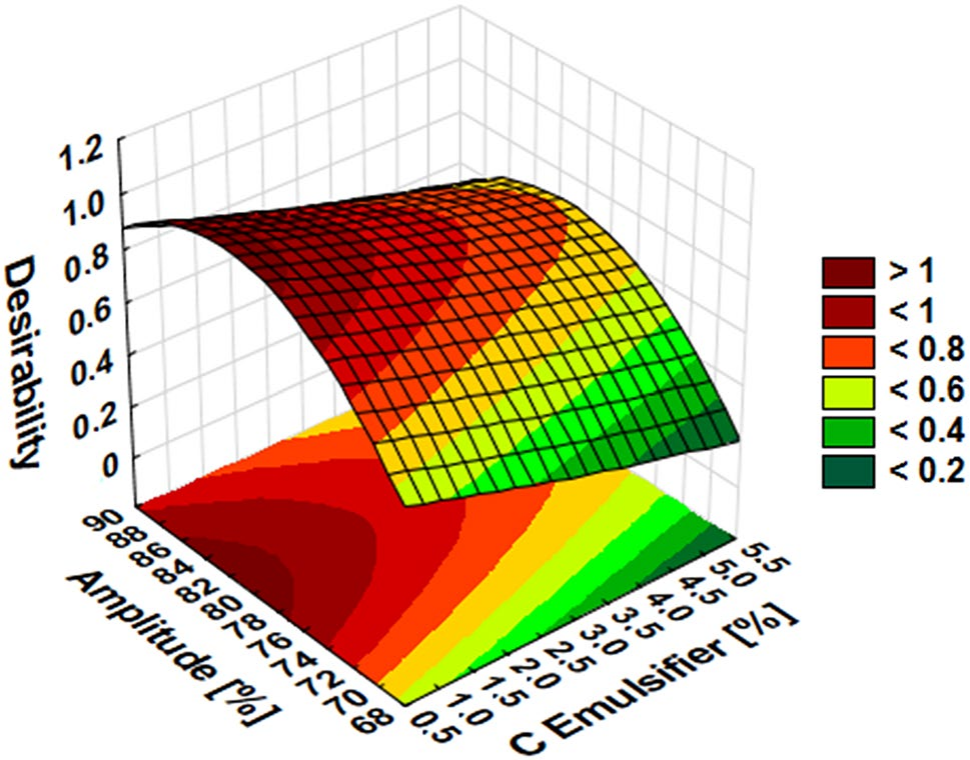
The saddle plot for desirability concerning emulsifier concentration and amplitude.

Because the vast majority of preparations made to plan the experiment were characterized by a particle size of less than 200 nm, it was decided to select the optimal compositions based on the data obtained from the saddle diagram showing the effect of emulsifier concentration and ultrasound amplitude (Fig. 4), and therefore, the input parameters having the greatest statistically significant impact on the viscosity of the nanoemulsion (Fig. 1b). Figure 4 shows that amplitude in the 76–89% range and an emulsifier in a concentration of up to 2% should be used to obtain a nanoemulsion with the lowest viscosity, according to statistically significant Pareto parameters (Fig. 1b).

According to the relationships presented in the approximation profiles (Figs. 2 and 3), the size of the nanoemulsion particles decreased with the increase in the emulsifier concentration, in contrast to the viscosity. A further increase in the concentration of the emulsifier resulted in a slight increase in the particle size of the internal phase of the nanoemulsion and a further increase in viscosity (Table 2).

### Metabolic activity of maize and barnyard grass seedlings treated with nanoemulsions

Thermal power is emitted by plant tissue during germination and growth and is directly proportional to the metabolic activity of the plant tissue.

**Figure 5:**
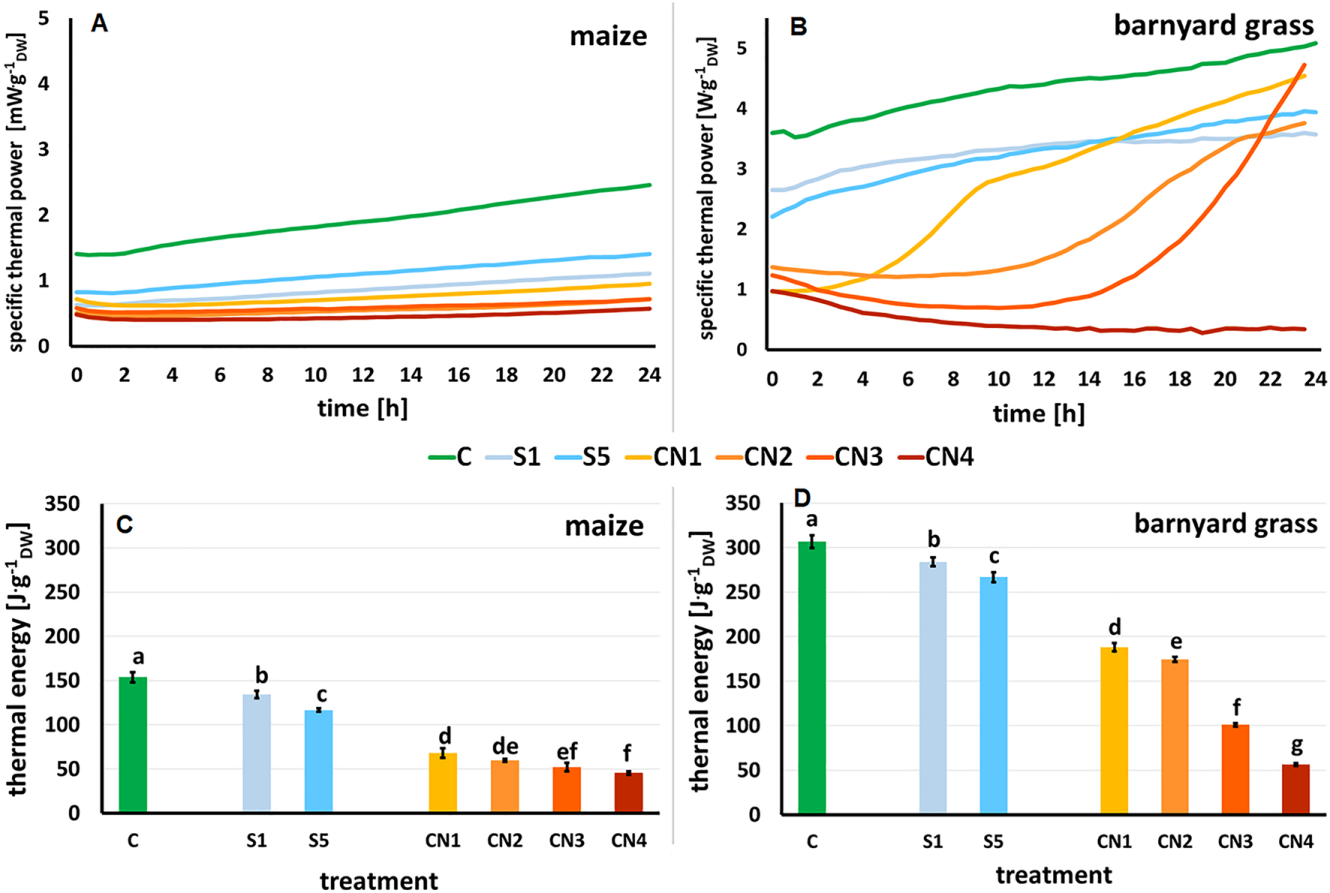
The specific thermal power curves of the maize (**A**) and barnyard grass (**B**) seedlings growing on the surfactants (S1, 1% v/v, light blue line and S5, blue line), nanoemulsions with caraway oil (CN1, 1% v/v, yellow line; CN2, 10% v/v, light orange line; CN3, 2% v/v, orange line; CN4, 5% v/v, dark orange line) and water [(**C**), green line]. The total heat emitted by maize (**C**) and barnyard grass (**D**) seedlings growing on the same treatments. Mean values ± SD are shown. Different letters indicate statistically significant differences at *p* ≤ 0.05 according to Duncan's test, *n* = 10.

The shape of maize thermal power curves showed a linear increase in all treatments (Fig. 5A). After 24 h of measurements, the highest value of specific thermal power was noted for control seedlings (C) and reached 2.46 mW·g_dry weight_^−1^ (g_DW_^−1^). At the end of the measurements, the values 1.41 and 1.11 mW·g_DW_^−1^ were recorded for seedlings growing on surfactants S1 and S5, respectively. Applying caraway oil to the nanoemulsions reduced the thermal power values, which were 0.95 mW·g_DW_^−1^ for CN1, 0.72 mW·g_DW_^−1^ for CN2 and CN3, and 0.58 mW·g_DW_^−1^ for CN4 (Fig. 5A).

In the case of barnyard grass, the thermal power curve for the control seedlings (C) increased linearly and reached the value of 5.09 mW·g_DW_^−1^ after 24 h (Fig. 5B). The thermal power curves for seedlings growing on surfactants S1 and S5 had a similar shape and amounted to 3.57 mW·g_DW_^−1^ for S1 and 3.94 mW·g_DW_^−1^ for S5 at 24 h, respectively. CNs differentially affected thermal power curves along 24 h. The trend of this parameter increased up to 4.54 mW·g_DW_^−1^ in seedlings treated with CN1, described a parabola in CN2 and CN3 seedlings with a final value of 3.76 mW·g_DW_^−1^ and 4.73 mW·g_DW_^−1^, respectively, and reduced the value up to 0.34 mW·g_DW_^−1^ in the CN4 treatment (Fig. 5B).

The highest value of the thermal energy was found for the control seedlings (C), ~ 154 J·g_DW_^−1^ for maize and ~ 307 J·g_DW_^−1^ for barnyard grass (Fig. 5C, D). Surfactants S1 and S5 reduced the thermal energy by 13% and 24%, respectively, in maize (Fig. 5C) and by 7.5% and 13%, respectively in barnyard grass, (Fig. 5D), when compared to the control.

In maize and barnyard grass seedlings, thermal energy was significantly decreased under CNs treatments. The higher oil concentration in the CNs, the lower the value of thermal energy (Fig. 5C, D). Compared to the control, maize seedlings' thermal energy decreased by 56%, 61%, 66%, and 70% in CN1, CN2, CN3, and CN4, respectively (Fig. 5C). However, compared to the control, barnyard grass seedlings' thermal energy decreased by 39%, 43%, 67%, and 82% when sprayed with CN1, CN2, CN3, and CN4, respectively (Fig. 5D).

### FT-Raman spectroscopy of maize and barnyard grass seedlings treated with nanoemulsions

**Figure 6:**
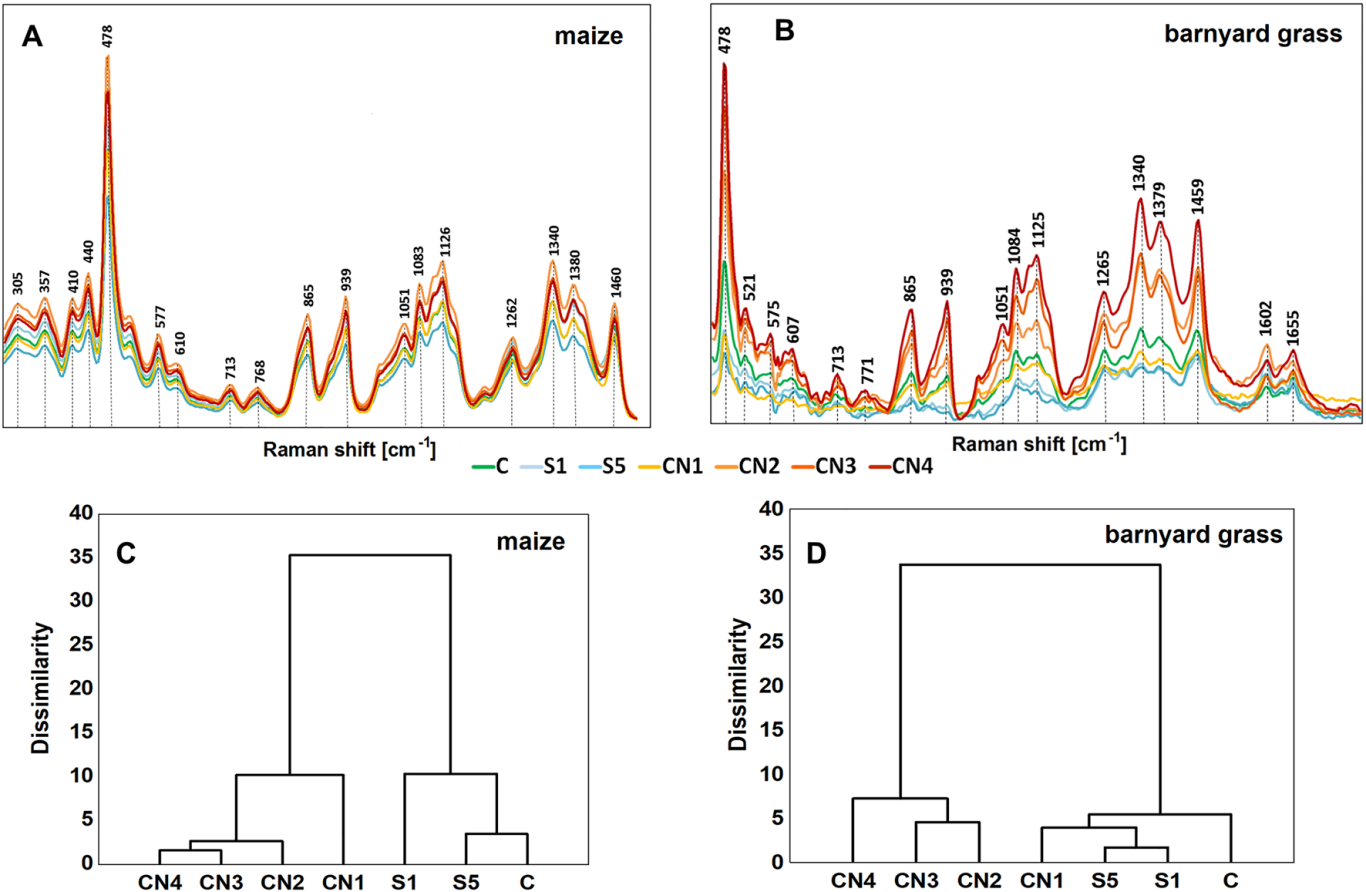
FT-Raman spectra show the chemical composition of seedling's endosperm of maize (**A**) and barnyard grass (**B**) that were growing on the surfactants (S1, light blue line, and S5, blue line), nanoemulsions with caraway oil (CN1, yellow line; CN2, light orange line; CN3, orange line; CN4, dark orange line) and water (C, green line). The mean values were based on six repetitions. Hierarchical cluster analysis of the FT-Raman spectra of the maize (**C**) and barnyard grass (**D**). Surfactants S1 (1% v/v) and S5 (10% v/v), nanoemulsions with caraway oil CN1 (1% v/v), CN2 (1.5% v/v), CN3 (2% v/v), CN4 (5% v/v), and water (C—control).

All analyzed samples showed spectra with numerous peaks reflecting their chemical composition. The pattern of peaks was similar in all treatments but had different intensities. However, we selected patterns representing spectral region between 300 and 1800 cm^−1^ for the analysis (Fig. 6A, B). This region corresponds to the presence of carbohydrates in the endosperm of maize and barnyard grass (Fig. 6A, B, respectively), which are storage substances. On the spectra are visible distinct bands, which correspond to vibrations of functional groups in carbohydrates (300–600 cm^−1^—endocyclic and exocyclic deformations; 800–1150 cm^−1^, so-called: "glucose fingerprint"), amylose and amylopectins (1340 cm^−1^ and 1380 cm^−1^), carbohydrates and lipids (1460 cm^−1^—a combination of the C-H deformation vibrations of lipids and starch), and lignins (1604 cm^−1^—C–H stretching of the aromatic ring) (Fig. 6A, B; Table 3). The most intense band (478 cm^−1^; Fig. 6A, B) is one of the dominating and important skeletal vibration modes of the pyranose ring, which depicts the degree of polymerization in polysaccharides and can be used as a marker to identify the presence of starch in the plant samples. The remaining bands visible on the spectra are related to carbohydrates (Table 3),

**Table 3:** Vibrational bands and their assignments for maize and barnyard grass.

Band	Vibrational mode	Assignment
305	Skeletal modes of pyranose ring	Carbohydrates
357	Skeletal modes of the pyranose ring	Carbohydrates
410	δ(C–C–O) + δ(C–C–C)	Carbohydrates
440	δ(C–C–O) + δ(C–C–C) skeletal modes of pyranose ring	Carbohydrates
478	CCO and CCC deformations; related to glycosidic ring skeletal deformations δ(C−C−C) + τ(C−O) scissoring of C−C−C and out-of-plane bending of C−O	Carbohydrates (starch)
521	S−S gauche–gauche–trans	Protein
577 (575)	δ(C−C−O) + τ(C−O)	Carbohydrates
610 (607)	δ(C−C−C)	Carbohydrates
713	δ(C−C−O) related to glycosidic ring skeletal deformations	Carbohydrates
768 (771)	δ(C−C−O)	Carbohydrates
865	δ(C−C−H) + δ(C−O−C) glycosidic bond; anomeric region	Carbohydrates
939	δ(C−O−C) + δ(C−O−H) + ν(C−O) α-1,4 glycosidic linkages	Carbohydrates
1051	ν(C–O) + ν(C–C) + δ(C–O–H)	Cellulose, lignin
1083 (1084)	ν(C−O) + ν(C−C) + δ(C−O−H)	Carbohydrates
1126 (1125)	ν(C−O) + ν(C−C) + δ(C−O−H)	Carbohydrates
1262 (1265)	δ(C−C−H) + δ(O−C−H) + δ(C−O−H)	Carbohydrates
1340	ν(C−O); δ(C−O−H)	Carbohydrates
1380 (1379)	δ(C−O−H)—coupling of the CCH and COH deformation modes	Carbohydrates
1460 (1459)	δ(CH_2_) + δ(CH_3_); δ(CH) + δ(CH_2_) + δ(C−O−H) CH, CH_2_ and COH deformations	Lipids, carbohydrates
1602	ν(C–C)ring + σ(CH)	Lignin
1655	ν(C=O) stretching	Amide I α-helix

Seedlings treated with CN2, CN3, and CN4 showed higher amount of glucose (higher intensity in 800–1150 cm^−1^ bands) compared to control (C) and surfactant (S1 and S5) seedlings (Fig. 6A, B).

For the endosperm of maize seedlings, the cluster analysis (similarity) was performed in the band range from 280 to 1500 cm^−1^ and for the endosperm of barnyard grass seedlings in the band range from 450 to 1800 cm^−1^ (Fig. 6A, B, respectively). The cluster analysis rendered two different groups for both maize and barnyard grass (Fig. 6C, D). For maize, the first group included control seedlings and those grown with surfactants (S1 and S5). In the second group, seedlings were treated with nanoemulsions with caraway oil at all tested concentrations—CN1, CN2, CN3, and CN4 (Fig. 6C). In the case of barnyard grass, control, surfactants (S1 and S5) and caraway oil at the lowest concentration (CN1) treatments grouped together and apart from CN2, CN3, and CN4 treatments (Fig. 6D).

### Herbicidal effects of leaf-sprayed nanoemulsions on maize and barnyard grass

Maize was less sensitive to damage caused by CNs than barnyard grass (Table 4). The oil doses in CNs treatments causing significant damage to aboveground parts of maize were estimated to be out of the assayed range; hence the results have a large standard error value. At the same time, these results indicate that the CNs with caraway oil at concentration up to 10% were harmless to maize. In contrast, for barnyard grass, 10% damage (ED_10_) was observed at CN3 (containing 2.0% of oil) (), 50% damage (ED_50_) was estimated at CN4 (containing a dose of 5.0% oil), and 90% damage (ED_90_) was estimated for a CN dose out of the assayed range, with estimated 13.5% of oil content (Table 4).

**Table 4:** The ED (effective dose) % values (± standard error), representing the dose of caraway essential oil (CEO) causing 10, 50 or 90% of injuries on the aboveground parts of maize and barnyard grass, as calculated by the dose–response analysis.

ED value	Maize (%)	Barnyard grass (%)
ED 10	11.4 ± 34.6	2.0 ± 0.37
ED 50	17.5 ± 57.4	5.1 ± 3.63
ED 90	26.9 ± 38.7	13.4 ± 20.8

#### Chlorophyll *a* fluorescence of maize and barnyard grass sprayed with nanoemulsions

**Figure 7:**
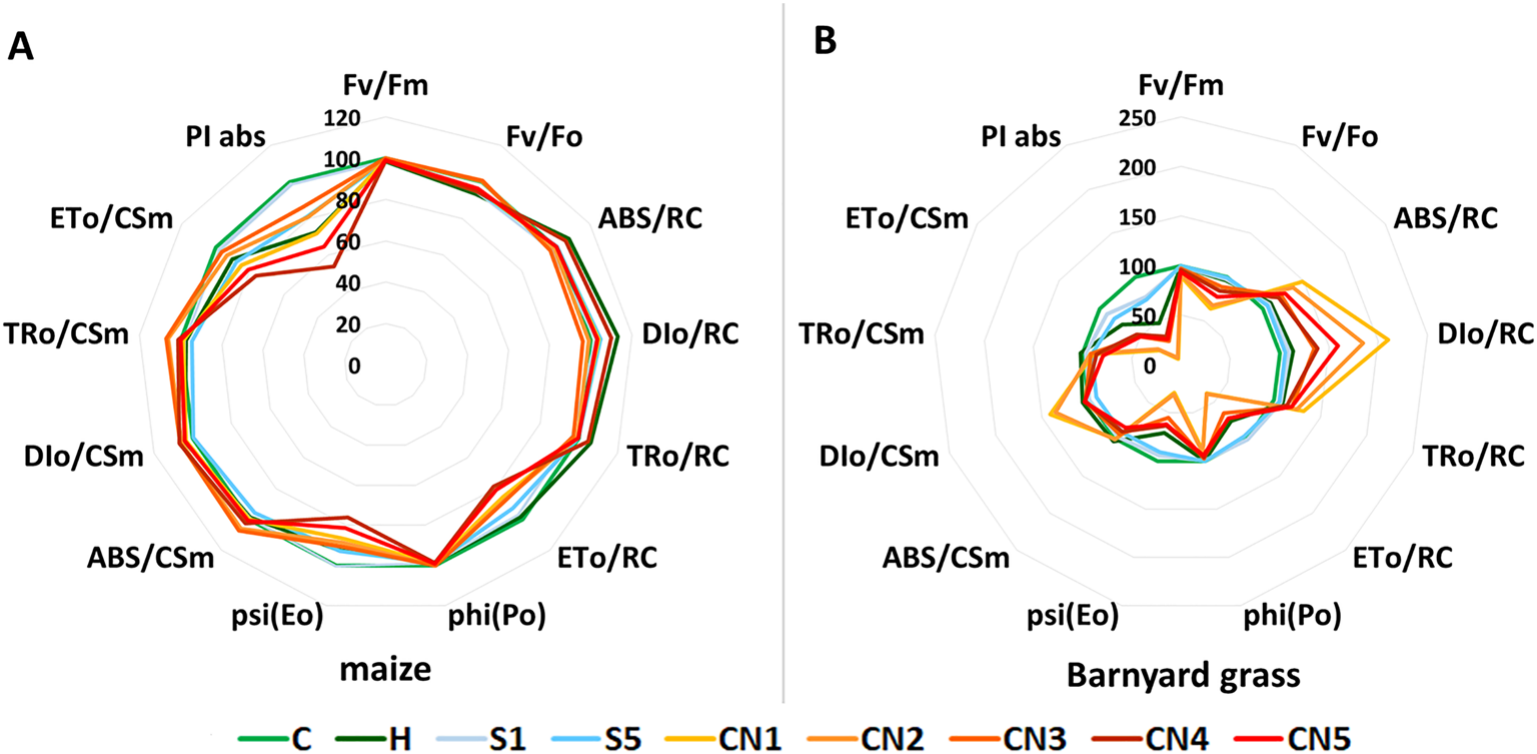
Values of selected Chl *a* fluorescence parameters of maize (**A**) and barnyard grass (**B**). C-water control, H-herbicide, surfactants S1 (1% v/v) and S5 (10% v/v), nanoemulsions with caraway oil CN1 (1% v/v), CN2 (1.5% v/v), CN3 (2% v/v), CN4 (5% v/v), and CN5 (10% v/v).

Based on the fast-kinetic chlorophyll *a* fluorescence the efficiency of the PSII was described and shown on the spider graphs, where the values for the control plants (C) are presented as 100% (Fig. 7A, B).

In the case of maize, most of the calculated parameters of PSII efficiency suffer slightly variations after spraying with surfactant only (S) or nanoemulsions with caraway oil (CNs) (Fig. 7A, Table 5). The F_v_/F_m_ and F_v_/F_0_ parameters did not vary among control (C), S and CNs sprayed plants, but were significantly reduced by 1.5% and 6.5%, respectively, in herbicide-sprayed (H) plants compared to control. Among the parameters that describe the specific energy fluxes calculated per reactive center (RC), herbicide (H) increased ABS/RC and DI_0_/RC parameters by 7.6% and 13.3%, respectively, compared to control (C). Treatments did not affect TR_0_/RC. A significant decrease in ET_0_/RC was observed after spraying with S5 (7.3%) and all CNs (21.2% for CN4). Parameter Phi (Po) was significantly reduced by 7.6% only for H compared to C. However, CNs treatments reduced the Psi (Eo) value compared to the control. The decrease ranged from 9.5% for CN3 to 23.8% for CN4. The parameters connecting with the phenomenological fluxes per cross-section (CS_m_) were reduced mainly by CNs treatments. Significant increases in ABS/CS_m_ and TR_0_/CS_m_ were observed for the CN2 and CN3 treatments. CN2 increased both parameters by 6% and CN3 by 7.1% ABS/CS_m_ and 7% TR_0_/CS_m_. Spraying with CN2, CN3, and CN4 caused a significant increase in DI_0_/CS_m_ by 5.9%, 6.5%, and 7%, respectively, compared to the control. Compared to C, parameter ET_0_/CS_m_ was strongly reduced in S5 by 12.1%, CN1 by 15%, CN4 by 23.8%, and CN5 by 19%. Stress indicator (PI) was significantly reduced by H (27.5%), CN1 (28%), CN4 (46.1%), and CN5 (35.5%) compared to C.

**Table 5:** Chl *a* fluorescence parameters for control and treated with herbicide, surfactants and CNs maize leaves.

Parameters	Maize								
	C	H	S1	S5	CN1	CN2	CN3	CN4	CN5
Fv/Fm	0.786^a^	0.775^b^ (−1.5)	0.777^ab^ (−1.2)	0.778^ab^ (−1.0)	0.779^ab^ (−0.9)	0.787^a^ (+ 0.1)	0.788^a^ (+ 0.2)	0.778^ab^ (−1.1)	0.780^ab^ (−0.8)
Fv/Fo	3.697^ab^	3.456^c^ (-6.5)	3.499^bc^ (-5.4)	3.518^abc^ (-4.8)	3.545^abc^ (-4.1)	3.708^ab^ (+ 0.3)	3.726^a^ (+ 0.8)	3.507^abc^ (-5.1)	3.559^abc^ (-3.7)
ABS/RC	1.789^bc^	1.926^a^ (+ 7.6)	1.750^c^ (-2.2)	1.805^abc^ (+ 0.9)	1.795^abc^ (+ 0.3)	1.774^bc^ (-0.9)	1.737^c^ (+ 2.9)	1.894^ab^ (+ 5.9)	1.798^abc^ (+ 0.5)
DIo/RC	0.383^bc^	0.435^a^ (+ 13.3)	0.392^abc^ (+ 2.2)	0.402^abc^ (+ 4.7)	0.398^abc^ (+ 3.8)	0.380^bc^ (-1.0)	0.369^c^ (-3.8)	0.422^ab^ (+ 10.0)	0.396^abc^ (+ 3.3)
TRo/RC	1.406^ab^	1.491^a^ (+ 6.1)	1.358^b^ (-3.4)	1.403^ab^ (-0.2)	1.396^ab^ (-0.7)	1.394^ab^ (-0.8)	1.369^b^ (-2.7)	1.472^a^ (+ 4.7)	1.401^ab^ (-0.3)
ETo/RC	0.810^a^	0.797^ab^ (-1.6)	0.784^ab (^-3.1)	0.750^bc^ (-7.3)	0.693^de^ (-14.4)	0.706^cd^ (-12.8)	0.709^cd^ (-12.5)	0.638^e^ (-21.2)	0.655^de^ (-19.1)
Phi (Po)	0.786^ab^	0.775^c^ (-1.4)	0.777^bc^ (-1.2)	0.778^abc^ (-1.0)	0.779^abc^ (-0.9)	0.787^ab^ (+ 0.1)	0.788^a^ (+ 0.2)	0.778^abc^ (-1.1)	0.780^abc^ (-0.8)
Psi (Eo)	0.578^a^	0.536^ab^ (-7.4)	0.580^a^ (+ 0.3)	0.537^ab^ (-7.2)	0.500^bc^ (-13.5)	0.512^bc^ (-11.5)	0.523^bc^ (-9.5)	0.441^d^ (-23.8)	0.471^cd^ (-18.5)
ABS/CSm	1347^cd^	1332^cd^ (-1.2)	1337^cd^ (-0.7)	1289^d^ (-4.3)	1344^cd^ (-0.2)	1428^ab^ (+ 6.0)	1447^a^ (+ 7.1)	1382^bc^ (+ 2.5)	1357^c^ (+ 0.8)
DIo/CSm	287^b^	299^ab^ (+ 4.2)	298^ab^ (+ 3.9)	286^b^ (-0.5)	297^ab^ (+ 3.4)	304^a^ (+ 5.9)	306^a^ (+ 6.5)	307^a^ (+ 7.0)	299^ab^ (+ 4.0)
TRo/CSm	1060^cd^	1033^cd^ (-2.6)	1039^cd^ (-2.0)	1004^d^ (-5.4)	1048^cd^ (-1.2)	1124^ab^ (+ 6.0)	1137^a^ (+ 7.2)	1075^bc^ (+ 1.3)	1059^cd^ (-0.1)
ETo/CSm	617^a^	556^abcd^ (-9.8)	604^ab^ (-2.0)	542^bcd^ (-12.1)	524^cde^ (-15.0)	575^abc^ (-6.7)	595^abc^ (-3.6)	470^e^ (-23.8)	499^de^ (-19.0)
PI	2.963^a^	2.149^bc^ (-27.5)	2.916^a^ (-1.6)	2.409^ab^ (-18.7)	2.134^bc^ (-28.0)	2.397^ab^ (-19.1)	2.561^ab^ (-13.6)	1.597^c^ (-46.1)	1.911^bc^ (-35.5)

Mean values marked with the same letters did not differ significantly at p ≤ 0.05 according to Duncan’s test, n = 15. The percentage values of changes compared to the control plants (taken as 100%) are given in parenthesis.

The parameters of phenomenological energy fluxes: the energy absorption by the antenna pigments (ABS/CSm), the amount of energy that was trapped in the reaction center (TRo/CSm), the energy flux for the electron transport (ETo/CSm), and the dissipation of energy as heat (DIo/CSm) where CS is the sample cross-section. The same parameters were also calculated for the reaction centre (RC) and named specific energy fluxes. The maximum quantum yield of the photosystem II primary photochemistry (Fv/Fm ratio), the maximum quantum yield of primary photochemistry (Fv/F0), the PSII performance index (PI).

For barnyard grass, spraying with caraway oil at any concentration (CN1-5) caused a significant decrease in the F_v_/F_m_ and F_v_/F_0_ parameters, compared to C, H, S1, and S5 (Fig. 7B, Table 6). The inhibition ranged from 2.6% in CN3 to 10.7% in CN1 for F_v_/F_m_ and from 11.3% in CN3 to 36.3% in CN1 for F_v_/F_0_ when compared to control (Fig. 7B, Table 6). Spraying of all CNs also significantly changed the value of parameters describing the specific energy fluxes calculated per reactive center (RC) compared to C, H, S1, and S5 (Fig. 7B, Table 6). The application of CN1 resulted in the highest increase value of 48.3% for ABS/RC, 110.7% for DI_0_/RC, and 32.4% for TR_0_/RC, and the largest decrease of 61.3% for ET_0_/RC. However, CN4 caused the lowest increment in these parameters. Compared to C, significant reductions in Phi (Po) parameters were observed after CNs application, from 2.6% for CN3 to 10.7% for CN1. Moreover, Psi (Eo) was significantly decreased in H, S, and all CNs treatments compared to C. Among the parameters described in the phenomenological energy fluxes per excited cross-section (CS_m_) (Fig. 7B, Table 6), ABS/CS_m_ was significantly reduced by S5, CN3, CN4, and CN5 compared to C. In addition, all CNs and S5 treatments resulted in a significant decrease in TR_0_/CS_m_ and ET_0_/CS_m_. The largest decrease was recorded for TR_0_/CS_m_ in CN5 (20.4%) and for ET_0_/CS_m_ in CN1(73.9%). Spraying with CN1 and CN2 caused a significant increase in DI_0_/CS_m_ value by 41.8% and 35.9%, respectively. However, the use of S5 resulted in a reduction of this parameter by 8.3% compared to the control. The performance index (PI) used as an indicator of stress was strongly reduced by all the treatments compared to C (Fig. 7B, Table 6). Surfactants showed the smallest decreases (22.7% in S1 and 25.3% in S5). On the contrary, H reduced PI by 52.4%, and CNs reduced this parameter by 67.9% in CN4 to 93.4% in CN1.

**Table 6:** Chl *a* fluorescence parameters for control and treated with herbicide, surfactants, and CNs barnyard grass leaves.

Parametrs	Barnyard grass								
	C	H	S1	S5	CN1	CN2	CN3	CN4	CN5
Fv/Fm	0.797^a^	0.791^ab^ (-0.8)	0.793^ab^ (-0.5)	0.796^a^ (-0.1)	0.712^e^ (-10.7)	0.727^e^ (-8.7)	0.776^bc^ (-2.6)	0.765^ cd^ (-4.0)	0.749^d^ (-6.0)
Fv/Fo	3.945^a^	3.791^a^ (-3.9)	3.844^a^ (-2.6)	3.919^a^ (-0.6)	2.514^d^ (-36.3)	2.684^d^ (-32.0)	3.500^b^ (-11.3)	3.325^bc^ (-15.7)	3.061^c^ (-22.4)
ABS/RC	1.790^e^	1.969^de^ (+ 10.0) )	1.923^e^ (+ 7.4) )	1.895^e^ (+ 5.9) )	2.654^a^ (+ 48.3) )	2.460^b^ (+ 37.4) )	2.199^c^ (+ 22.9) )	2.131^ cd^ (+ 19.1) )	2.276^c^ (+ 27.2) )
DIo/RC	0.364^e^	0.413^e^ (+ 13.6)	0.398^e^ (+ 9.5)	0.386^e^ (+ 6.0)	0.766^a^ (+ 110.7)	0.673^b^ (+ 85.0)	0.493^d^ (+ 35.5)	0.504^d^ (+ 38.5)	0.579^c^ (+ 59.2)
TRo/RC	1.426^e^	1.556^de^ (+ 9.1)	1.524^de^ (+ 6.9)	1.510^de^ (+ 5.9)	1.888^a^ (+ 32.4)	1.787^ab^ (+ 25.3)	1.706^bc^ (+ 19.6)	1.627^ cd^ (+ 14.1)	1.697^bc^ (+ 19.0)
ETo/RC	0.803^a^	0.609^b^ (-24.2)	0.811^a^ (+ 0.9)	0.772^a^ (-3.9)	0.311^d^ (-61.3)	0.311^d^ (-61.2)	0.518^c^ (-35.5)	0.579^bc^ (-28.0)	0.578^bc^ (-28.1)
Phi (Po)	0.797^a^	0.791^ab^ (-0.8)	0.793^ab^ (-0.5)	0.796^a^ (-0.1)	0.712^e^ (-10.7)	0.727^e^ (-8.7)	0.776^bc^ (-2.6)	0.765^cd^ (-4.0)	0.749^d^ (-6.0)
Psi (Eo)	0.566^a^	0.399^c^ (-29.5)	0.533^ab^ (-5.9)	0.514^b^ (-9.3)	0.165^e^ (-70.9)	0.175^e^ (-69.1)	0.312^d^ (-44.9)	0.358^ cd^ (-36.9)	0.351^ cd^ (-38.0)
ABS/CSm	1264^a^	1301^a^ (+ 2.9)	1231^ab^ (-2.6)	1148^c^ (-9.2)	1262^a^ (-0.1)	1275^a^ (+ 0.9)	1186^bc^ (-6.1)	1133^cd^ (-10.4)	1070^d^ (-15.4)
DIo/CSm	255^b^	272^b^ (+ 6.5)	255^b^ (-0.3)	234^c^ (-8.3)	362^a^ (+ 41.8)	347^a^ (+ 35.9)	265^b^ (+ 3.7)	265^b^ (+ 3.6)	267^b^ (+ 4.5)
TRo/CSm	1008^a^	1029^a^ (+ 2.0)	976^ab^ (-3.2)	914^bc^ (-9.4)	900^c^ (-10.7)	928^bc^ (-7.9)	921^bc^ (-8.6)	868^c^ (-13.9)	803^d^ (-20.4)
ETo/CSm	573^a^	413^c^ (-27.9)	520^ab^ (-9.2)	472^b^ (-17.7)	149^e^ (-73.9)	163^e^ (-71.5)	290^d^ (-49.3)	312^d^ (-45.5)	286^d^ (-50.1)
PI	2.990^a^	1.423^c^ (-52.4)	2.309^b^ (-22.7)	2.235^b^ (-25.3)	0.197^e^ (-93.4)	0.238^e^ (-92.0)	0.802^d^ (-73.2)	0.960^d^ (-67.9)	0.882^d^ (-70.5)

Mean values marked with the same letters did not differ significantly at *p* ≤ 0.05 according to Duncan’s test, *n* = 15. The percentage values of changes compared to the control plants (taken as 100%) are given in parenthesis.

The parameters of phenomenological energy fluxes: the energy absorption by the antenna pigments (ABS/CSm), the amount of energy that was trapped in the reaction center (TRo/CSm), the energy flux for the electron transport (ETo/CSm), and the dissipation of energy as heat (DIo/CSm) where CS is the sample cross-section. The same parameters were also calculated for the reaction centre (RC) and named specific energy fluxes. The maximum quantum yield of the photosystem II primary photochemistry (Fv/Fm ratio), the maximum quantum yield of primary photochemistry (Fv/F0), the PSII performance index (PI).

#### Relative water content of maize and barnyard grass sprayed with nanoemulsions

**Figure 8:**
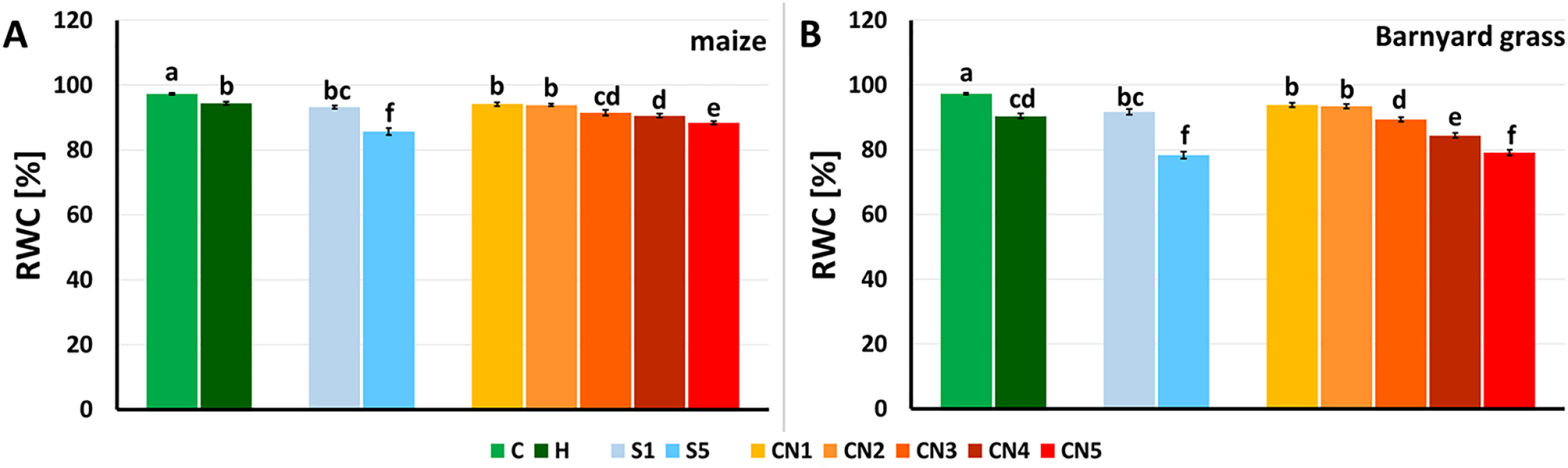
Relative water content (RWC) of maize (**A**) and barnyard grass (**B**) that were growing on the surfactants S1 (1% v/v) and S5 (10% v/v), nanoemulsions with caraway oil CN1 (1% v/v), CN2 (1.5% v/v), CN3 (2% v/v), CN4 (5% v/v), and CN5 (10% v/v), herbicide (H) and water (C). Mean values ± SD are shown. Different letters indicate statistically significant differences at *p* ≤ 0.05 according to Duncan's test, *n* = 10.

72 h after spraying with H, surfactants (S1, S5), or nanoemulsions with caraway oil (CNs), the relative water content (RWC) statistically significantly decreased compared to the C in maize (Fig. 8A) and in barnyard grass leaves (Fig. 8B).

In maize leaves, H, CN1, CN2, and S1 were a homogeneous group that had the smallest effect on RWC value compared to the C. The lowest RWCs were found for S5 and CN5 treatments, which reduced the RWC values by 12% and 9%, respectively, compared to C (Fig. 8A).

For barnyard grass, RWC was slightly reduced in CN1 and CN2 (decrease by less than 4%) and SN1 (decrease by nearly 6%) compared to C. Spraying plants with H, CN3, and CN4 reduced RWC by 7%, 8%, and 13% respectively in comparison to C. The largest changes in RWC were observed in S5 and CN5 with values of 18% or more lower than those in control. (Fig. 8B).

## Discussion

The biological properties of caraway essential oil are determined by its two main volatiles, i.e., a monoterpene ketone carvone in the form of (S)-(+)-isomer (relative configuration: D) and monoterpene hydrocarbon—(R)-(+)-D-limonene (relative configuration: D), of citrus-like scent^19^. What is typical for caraway oil is that the other detected volatiles usually compose less than a trace amount of essential oil. It can be assumed that the studied caraway oil biological properties were related to its main compounds, which were present in a 1:0.55 ratio of carvone to limonene. Among these two compounds, carvone is much more toxic than limonene, which was confirmed by Jop et al.^27^, who studied the phytotoxic potential of a fractionated mixture of caraway-origin carvone and limonene in different proportions. They found that a mixture in which carvone dominated caused a total inhibition of wheat, wild oat, and chamomile germination. Contrary, when limonene dominated the mixture, wheat and wild oat germinated to a higher extent.

Generally, in the case of essential oils, the phytotoxic potential of their compounds is not enough, and a proper formulation e.g. nanoemulsion is essential to improve their biological activity and selectivity. Nanoemulsions can also decrease the side effects of essential oils^15^. Our previous work showed that peppermint oil forms a stabile nanoemulsion with Eco Tween 80^32^. In the present study, the same emulsifier formed a stabile nanoemulsion with the caraway essential oil, up to *ca*. 2% of the oil concentration. In designing the caraway-loaded nanoemulsion composition, the desired properties were the smallest droplet size, polydispersity index, and viscosity. The significant effect of both linear and a square functions of amplitude proves a higher influence of this parameter on nanoemulsion's viscosity^16^.

We studied the biological effect of nanoemulsions with caraway oil (CNs) in comparison to Eco Tween 80 (S) and herbicide (H) on the metabolism of maize and barnyard grass seedlings. Isothermal calorimetry was employed to allow direct measurements of thermal activities (thermal power) and, thus, energies involved in the biological processes of the seedlings. This method was successfully used to determine the effect of herbicides^33,34^, essential oils^16,47^, or allelopathic compounds^31,32^ on general plant metabolism. The energy emitted during life processes is directly related to metabolic activity and shows the reaction of the plant to stress factors^48^. Moreover, the metabolic activity of tissues is directly proportional to the total amount of energy emitted by them^35^. The highest values of emitted thermal energy characterized control maize and barnyard grass seedlings. Treatment with S and CNs at all concentrations decreased metabolic activity in both species through a lower amount of emitted energy. The higher the concentration of caraway oil in nanoemulsions, the lower the amount of released energy. Thus, the nanoemulsion with 5% of the caraway oil inhibited the growth of maize and barnyard grass seedlings. These results are consistent with our previous work, where we showed that the nanoemulsion with 5% peppermint oil also caused the highest inhibition of growth of these species^16^. Using the FT-Raman spectroscopy method, we examined the effect of surfactants and nanoemulsions with caraway oil of various concentrations on the chemical composition of the endosperm of maize and barnyard grass seedlings. It is a non-destructive method that allows the determination of changes in the chemical composition of plant tissue under the influence of biotic and abiotic stresses^36,45^. Importantly, FT-Raman spectroscopy is sensitive even to small structural changes, which is useful in comparative studies^49^. Seed germination begins the process of water absorption, during which enzymes are activated. The storage materials are hydrolyzed into simpler components, which then serve as a source of energy for the embryo and the forming seedlings^50^. In both species studied by us, the storage materials are carbohydrates. For this reason, the bands originating from these compounds are visible in the spectra. The amount of a given chemical compound in the sample is proportional to the intensity of the band assigned to it in the spectrum^51^. FT-Raman showed an increase in monosaccharides (glucose) content derived from the hydrolysis of polysaccharides in the endosperm of maize and barnyard grass seedlings under the influence of nanoemulsions with the caraway oil at concentrations of 1.5%, 2%, and 5%. This shows that there is an acceleration of the decomposition of storage materials. A similar phenomenon was observed in the endosperm of maize and barnyard grass seedlings during growth in the presence of nanoemulsions with peppermint oil^16^, as well as during the growth of wild oats and maize in the presence of emulsions with essential hemp oil^52^ or during the growth of mustard and oilseed rape seedlings in the presence of herbal extracts^32^. We used Ward's cluster analysis to group the studied objects in terms of similarity in their chemical composition. The analysis confirmed the effect of nanoemulsion with caraway oil on the carbohydrates composition of maize and barnyard grass endosperm. In the case of maize, the first group consisted of plants treated with nanoemulsions with the caraway oil at each concentration used, compared to the second group of control and surfactant-only treated plants. In the case of barnyard grass metabolism, the lowest toxic effect was for plants treated with nanoemulsions containing 1% of caraway oil.

In our work, we also studied the effect of leaf-applied nanoemulsions with caraway oil on young plants of maize and barnyardgrass. Based on the dose–response test, we estimated the doses of nanoemulsions that cause 10, 50, and 90% of plant damages, observed mainly as necroses of leaf and stem tissues. We found that the nanoemulsions at doses containing up to 10% of the caraway oil were harmless to maize. In contrast, barnyard grass was much more sensitive to spraying with nanoemulsions, with 50% and 90% damage (ED_50_ and ED_90_) estimated at doses of nanoemulsions containing 5.1% and 13% of caraway oil, respectively. We find these results promising, as they show a possible selective application of the nanoemulsions with caraway oil in maize against barnyard grass. The observed damages of leaves were also confirmed by the measurements of RWC in leaves, which is an important indicator of water status in plants and useful as a stress indicator. The RWC reflects the balance between the water supply to the leaf tissue and the transpiration rate. Normal values of RWC are about 98%, while in severely desiccated and dying leaves, they are about 30–40%^53^. Essential oils, as well as allelopathic compounds, can cause a decrease in the RWC value. In the present study, the RWC values in all treatments were statistically significantly lower than the control. Moreover, with the increasing concentration of CNs, the RWC values of maize and barnyard grass leaves gradually decreased, reaching about 80% for barnyard grass and 85% for maize leaves. The largest decrease in RWCs compared to controls were observed for S5 (12%) and CN5 (9%) for maize and about 18% in both cases for barnyard grass. In previous work, we showed that nanoemulsions with peppermint oil reduced the RWC values of maize and barnyard grass leaves^16^. Based on our research, comparing the effect of the nanoemulsion with peppermint and caraway oils at the highest concentration (10%) on the RWC values, it can be concluded that peppermint oil affects the RWC value for maize to a similar extent as caraway oil. However, both nanoemulsions with peppermint oil or with the caraway oil significantly reduced the value of the RWC parameter in barnyard grass.

Following up, we also measured the fast kinetics of chlorophyll *a* fluorescence as a quick, non-invasive, and non-destructive method of measuring the efficiency of photosynthesis^54^. Such measurement provides information about the impact of stress factors on the plant's photosynthetic apparatus, thanks to which we can detect the effects of stress before visible external symptoms appear. It is believed that the parameter F_v_/F_m_ (the maximum quantum yield of the photosystem II primary photochemistry) may be an indicator of stress in plants^38^, which is also indicated by our results in barnyard grass, in which a significant decrease in the value of this parameter was observed after spraying with all nanoemulsions containing the caraway oil. The nanoemulsions with 1% and 1.5% of caraway oil registered F_v_/F_m_ values lower than 0.74 suggesting a photoinhibition^39^. However, no such changes were observed in maize, which may be related to the morphological structure of leaves. Maize leaves are thicker and covered with hairs and could be more resistant to penetration by biologically active compounds in caraway oil. Among the parameters describing the specific energy fluxes on the reactive reaction center (RC) of PSII, a significant increase in the parameters ABS/RC, DI_0_/RC, TR_0_/RC was observed in barnyard grass. In contrast, no significant changes were observed in maize compared to the control.

At the same time, the ET_0_/RC parameter describing the electron transport flux decreased in both species due to the inactivation of reaction center complexes (e.g., the inactivation of the oxygen-evolving system). Thus it could be directly related to a lower efficiency of the photosynthesis process^40^. Considering the phenomenological fluxes converted to the excited photosynthetic surface area of the sample (CS_m_), the largest changes were noted for the parameters DI_0_/CS_m_ and ET_0_/CS_m_ in the treatment of CN1 and CN2 compared to the control in barnyard grass. In maize, CNs spraying caused the greatest changes in the ET_0_/CS_m_ parameter values. In addition, both species showed a significant decrease in the Psi (Eo) parameter, which is also related to the electron transport (probability that a photon trapped by the PSII reaction center enters the electron transport chain)^55^. The results show that spraying leaves with nanoemulsions with the caraway oil mainly disturbed the transport of electrons, affecting the efficiency of photosynthesis and the condition of plants. These changes were observed to a greater extent in barnyard grass.

The performance index (PI) reflects the functionality of photosystems because it combines information on the number of reaction centers, the efficiency of energy trapping, and electron transport from PSII to the plastoquinone pool^37^. Our study confirmed that PI is a very good and sensitive indicator of the general condition of the plant after spraying with CN nanoemulsions. In maize, the highest reduction of this parameter, by 40–50% compared to the control, occurred when leaves were sprayed with nanoemulsions containing 5% and 10% of the caraway oil. However, it should be emphasized that applying nanoemulsion with 1% of the oil affected the PI to the same extent as the synthetic herbicide (approximately 25% lower than the control plants). A strong reduction of PI occurred in barnyard grass sprayed with all nanoemulsions, but those with 1% and 1.5% of the oil decreased the values by more than 92%. The above results showed that barnyard grass is more susceptible to the leaf-sprayed nanoemulsions with the caraway oil than maize, which is consistent with the results obtained in the case of plants spraying with nanoemulsions containing peppermint oil^16^.

In summary, low doses of nanoemulsions with caraway oil (1% and 1.5%) and Eco Tween 80 as a biodegradable surfactant, can impair barnyard grass metabolism without a significant damage on maize, which suggest a potential selective control of this weed that is common in maize crops. As an effect of action of the nanoemulsions we observed a slowed-down seedling metabolism and photosynthetic targets. We conclude, that the nanoemulsions with caraway oil could be used as a post-emergence herbicide on early barnyard grass development. However, additional studies under field conditions are needed to validate the observed effect, also considering the nanoemulsions’ effects on non-target organisms and soil microbes.

## Data Availability

The datasets generated during and/or analysed during the current study are available from the corresponding author on reasonable request.
